# Survival of red blood cells after transfusion: processes and consequences

**DOI:** 10.3389/fphys.2013.00376

**Published:** 2013-12-18

**Authors:** Giel J. C. G. M. Bosman

**Affiliations:** Department of Biochemistry, Radboud University Medical CentreNijmegen, Netherlands

**Keywords:** aging, autoimmunity, erythrocyte, membrane, transfusion, vesicle

## Abstract

The currently available data suggest that efforts toward improving the quality of red blood cell (RBC) blood bank products should concentrate on: (1) preventing the removal of a considerable fraction of the transfused RBCs that takes place within the first hours after transfusion; (2) minimizing the interaction of the transfused RBCs with the patient's immune system. These issues are important in reducing the number and extent of the damaging side effects of transfusions, such as generation of alloantibodies and autoantibodies and iron accumulation, especially in transfusion-dependent patients. Thus, it becomes important for blood bank research not only to assess the classical RBC parameters for quality control during storage, but even more so to identify the parameters that predict RBC survival, function and behavior in the patient after transfusion. These parameters are likely to result from elucidation of the mechanisms that underly physiological RBC aging *in vivo*, and that lead to the generation of senescent cell antigens and the accumulation of damaged molecules in vesicles. Also, study of RBC pathology-related mechanisms, such as encountered in various hemoglobinopathies and membranopathies, may help to elucidate the mechanisms underlying a storage-associated increase in susceptibility to physiological stress conditions. Recent data indicate that a combination of new approaches *in vitro* to mimick RBC behavior *in vivo*, the growing knowledge of the signaling networks that regulate RBC structure and function, and the rapidly expanding set of proteomic and metabolomic data, will be instrumental to identify the storage-associated processes that control RBC survival after transfusion.

## Introduction

The final days of the erythrocyte's life are characterized by the appearance of an aging-specific removal signal. The signal is a neoantigen that is derived from the integral membrane protein band 3, and the removal is phagocytosis that is initiated by binding of autologous IgG and subsequent recognition by macrophages in the liver and possibly in the spleen (Kay, 1975; Safeukui et al., 2012). Since the seminal papers of Kay and coworkers (Kay, 1975, 1978, 1981, 1984; Kay and Bennett, 1982; Kay et al., 1983, 1988a; Safeukui et al., 2012) with the observations and experiments supporting and/or based on this concept, recognition of old erythrocytes by a physiological autoimmune reaction has been generally accepted as responsible for the physiological disappearance of erythrocytes after 120 days in the circulation (Clark, 1988). However, little progress has been made with respect to the identity of the molecular mechanism(s) leading to the formation of the aging or senescent cell-specific antigen. This is the more frustrating, since the erythrocyte has the potential of becoming a model for cellular aging, as it once was for membrane structure. Also, the experimental and conceptual framework that has arisen around this concept, has the potential of becoming instrumental in the generation of biomarkers of various diseases, and in the expansion of our knowledge of the regulation of cell morphology and metabolism.

The aging framework is commonly—and often implicitly—used to interpret the molecular events occurring during erythrocyte storage in blood bank conditions (Bosman et al., 2008a, 2010a). The link between aging *in vivo* and aging *in vitro* is extended by the study of hereditary anemias caused by increased erythrocyte removal. The resulting triangle has been given at least one extra dimension by recent data indicating that, at the current scientific and technical level, the truly relevant blood bank conditions are not those that determine erythrocyte survival in the blood bank, but those that affect function and survival after transfusion (Bosman et al., 2011). Yet another, new dimension is formed by the status of the erythrocyte-receiving patient, e.g., the activity of the immune system and/or the spleen, as an effector of the survival of the transfused erythrocytes (Gould et al., 2007; Dinkla et al., 2012a,b).

The present review starts with a summary of the currently available knowledge of the molecular structure, function and metabolism of the aging erythrocyte in the healthy individual. This summary is the starting point for a review of the data obtained *in vitro* and from patients with hereditary erythrocyte pathologies, based on the view that this may help to deduce the most likely molecular mechanism(s) leading to the aged phenotype. The resulting synthesis constitutes the framework for a discussion of the storage lesions, focussing on their impact on the survival of erythrocytes after transfusion.

## Characteristics of erythrocyte aging *in vivo*

An overview on the available data, strictly limited to those obtained by analysis of erythrocyte aging *in vivo* in healthy people, shows the following:

### Lifespan

The maximal lifespan of erythrocytes is 120 days, with a rather small variation of approximately 10 percent. This variation may be due to variations in methodology, such as the analysis of appearance and disappearance of metabolic labels from the circulation, and of the disappearance of erythrocytes labeled with various markers after autologous transfusion, or after transfusion of erythrocytes with differences in minor blood groups (Werre et al., 2004; Bosman et al., 2012a). Alternatively, the variability in maximal lifespan may also be due to inter-individual variations in erythrocyte homeostasis, as has become apparent especially in recent blood bank donor research (Wenk et al., 2011; Dinkla et al., 2013). The relatively small variation in lifespan observed in all studies, however, suggests a gradual, multi-step mechanism rather than a random, disastrous insult, as well as a very efficient removal process.

### Volume and density

With increasing time in the circulation, erythrocytes become smaller and more dense. A detailed analysis of these changes shows that, with age, erythrocytes loose 30% of their volume and 15–20% of their hemoglobin, whereas the hemoglobin concentration increases by 14%. This implies that, with age, erythrocytes lose proportionally more water than hemoglobin. Because the decrease in volume is larger than the decrease in surface area, the surface to volume ratio increases. This theoretically positive effect on deformability is abolished by the increase in the hemoglobin concentration, and probably by a decrease in the membrane elasticity (Bosch et al., 1994). Using the percentage of glycated hemoglobin, HbA1c, as a marker of cell age in combination with cohort labeling, survival studies and hemocytometry, Werre and coworkers (Van der Vegt et al., 1985; Bosch et al., 1992, 1994) established that age-related purification of cell fractions on the basis of density alone has inherent restrictions. A similar conclusion can be drawn using another cell age marker, the 4.1a:4.1b ratio, which increases as the result of non-enzymatic deamidation (Mueller et al., 1987; Lutz et al., 1992; Ciana et al., 2004). The lighter fractions are strongly enriched for reticulocytes and young erythrocytes, but the dense fractions are much more heterogeneous with respect to cell volume and cell age. When counterflow centrifugation is followed by density centrifugation, the mean corpuscular volume decreases from 101 fl in the fraction containing the lightest and largest erythrocytes to 72 fl in the fraction with the most dense and smallest cells (Bosch et al., 1992). Combining the two separation techniques results in a considerable reduction in the cell volume-based distribution curves, an almost complete absence of overlap in the erythrocytograms of the lighter-larger and the denser-smaller fractions, and the largest difference in the percentage HbA1c between these fractions (Bosch et al., 1992). Thus, a combination of separation techniques based on volume and density yields erythrocyte fractions with a greater difference in mean cell age than does separation on the basis of density or volume alone.

### Vesiculation

Based on observations in splenectomized individuals and on the analysis of subcellular blood fractions, it has been postulated that, under normal conditions, vesiculation is responsible for the aging-associated loss of hemoglobin. The striking resemblance between the hemoglobin composition of blood-borne vesicles and that of old erythrocytes, supports the conclusion that there is a continuous loss of hemoglobin in vesicles, which accelerates during the second half of the erythrocyte lifespan (Willekens et al., 2003). In the oldest erythrocytes of asplenic individuals, the decrease in hemoglobin is absent, concomitant with an increase in the absolute amounts of glycated and otherwise modified hemoglobin species (Willekens et al., 2003). Together with the previous observations that erythrocytes of patients without a functional spleen have an increased number of hemoglobin-containing vacuoles (Reinhart and Chien, 1988), and that there is a positive relation between the vacuole-containing erythrocytes and the percentage HbA1c (De Haan et al., 1988), these data suggest that hemoglobin-containing vesicles within old erythrocytes are removed from the erythrocytes in the spleen. However, it is not likely that vesiculation and vesicle removal occur only in the spleen. A rough calculation based on the vesicle concentrations measured in the blood (Willekens et al., 2008), combined with the kinetics of vesicle disappearance as measured in a rat model (Willekens et al., 2005), indicates that erythrocyte-derived vesicles are phagocytized almost directly after they are generated, even before they can reach the venous circulation. Immunological, biochemical and proteomic analysis of the vesicles generated *in vivo* suggests that their origin and removal are intimately interwoven with the erythrocyte aging process, and especially with breakdown of band 3 (Willekens et al., 2008; Bosman et al., 2008a, 2012b). The increase in erythrocyte-derived vesicles described in various pathological conditions, most of which are directly related to erythrocyte-specific abnormalities in hemoglobin or membrane proteins, suggests a disturbed aging process in these diseases (Kozuma et al., 2011; Mahfoudhi et al., 2012). However, it has become clear that vesicles may be generated by various mechanisms, emphasizing the need for extensive analysis of these vesicles and comparison with those generated in healthy individuals (Willekens et al., 2005, 2008; Bosman et al., 2010a, 2012b; Kozuma et al., 2011; Xiong et al., 2011; Mahfoudhi et al., 2012; Nantokomol et al., 2012).

### Removal signals

**Figure 1:**
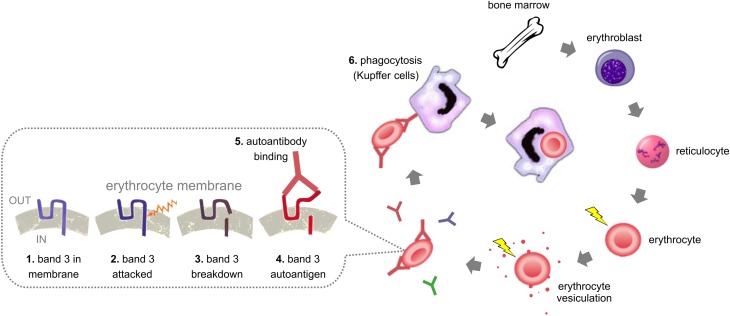
**The life and death of the erythrocyte**. The numbers refer to the putative steps in the generation and recognition of removal signals.

Alterations in band 3, as indicated by the presence of breakdown products and/or band 3-containing high-molecular-weight complexes, constitute the most consistent finding in the membranes of old erythrocytes (Kay, 2005; Pantaleo et al., 2008; Willekens et al., 2008; Bosman et al., 2010a,b). Together with the specificity of the senescent cell-specific IgG, this implies a band 3-derived antigen as the main factor responsible for the removal of old erythrocytes from the circulation. There is no convincing evidence for the involvement of phosphatidylserine (PS) in physiological removal of healthy, aged erythrocytes (Willekens et al., 2008; Franco et al., 2013). Recently, however, data have been presented indicating the involvement of the “self” antigen CD47 in phagocytosis of old erythrocytes (Burger et al., 2012). The observations that vesicles are enriched in IgG and band 3 breakdown products, and that most of them expose PS at their surface, have led to the theory that vesiculation serves to dispose damaged membrane patches (Willekens et al., 2008; Tissot et al., 2010). This mechanism would postpone the elimination of functional erythrocytes, and the fast removal of vesicles may prevent uncontrolled coagulation and inflammation (Xiong et al., 2011; Mahfoudhi et al., 2012). The current state of knowledge as described here is schematically depicted in Figure 1.

## Mechanisms of erythrocyte aging *in vivo*

So far, the data of the last decade have mostly confirmed and extended, but not deepened the picture sketched in the preceding paragraphs (Kay, 2005; Pantaleo et al., 2008; Franco et al., 2013). Incorporation of the vesicle characteristics into the aging process supports the putative early involvement of hemoglobin, and the central role of band 3 in the aging process (Salzer et al., 2008; Willekens et al., 2008; Tissot et al., 2010; Bosman et al., 2012b). The cytoplasmic domain of band 3 is a central mediator of the concentration of ATP, 2,3-DPG, NADH and NADPH (Messana et al., 1996; Chu et al., 2008; Rogers et al., 2009; Dzik, 2011). Thereby, aging-associated changes in band 3 connect changes in cell morphology and volume, deformability, and interaction between cytoskeleton and lipid bilayer with changes in the activity of the glycolytic and the pentose phosphate pathways, and possibly ion transport and release of ATP and NO as well. Binding of oxidatively modified hemoglobin, the so-called hemichromes, to the cytoplasmic domain of band 3 is likely to alter its conformation, and may thereby induce aggregation and/or increase its susceptibility to proteases. An aging-related increase in oxidation of membrane lipids and proteins may catalyze this process. Indeed, recent proteomic data show an aging-associated membrane recruitment of chaperone proteins, indicating denaturation and exposure of hitherto hidden protein domains (Bosman et al., 2012b). Also, metabolomic and biochemical data suggest a decrease in anti-oxidation defense, together with a decrease of glycolytic activity in erythrocytes aged *in vivo* (Bosman and Kay, 1988; Ghashghaeinia et al., 2012; D'Alessandro et al., 2013a). Alterations in the cytoplasmic domain of band 3 also affect binding of ankyrin, and loss of anchorage at this junction of the spectrin cytoskeleton to the lipid bilayer is the most likely cause of the formation of vesicles observed *in vivo*, and explains much of their protein composition (Sens and Gov, 2007; Gov et al., 2009). Erythrocyte aging in humans can be studied experimentally only *in vitro*, and physiological erythrocyte removal in mice is likely to differ too much from that in humans to expect that mouse studies will lead to new, relevant developments in this area (Khandelwal and Saxena, 2008). Therefore, support for the mechanisms that have been deduced from theory-driven or inventory-based comparisons of erythrocytes of various ages, has been sought in various erythrocyte-centered diseases. Detailed analysis of hemoglobinopathies, such as sickle cell disease and hemoglobin Köln, supports the relationship between hemoglobin deposition at the membrane, immunoglobulin binding, and also vesiculation (Kay et al., 1988b; Westerman et al., 2008). Immunoblot analysis of HbS-containing erythrocytes show sickling-associated and aged cell-like band 3 patterns (Bosman, 2004).

Altered vesiculation may underly the aberrant erythrocyte morphology caused by genetic abnormalities in band 3, ankyrin or spectrin leading to elliptocytosis. The role of the spleen in vesiculation is confirmed by the beneficial effect of splenectomy in these patients (An and Mohandas, 2008). It is noteworthy that the elliptocytosis-associated mutations are all located in the membrane domain of band 3, illustrating the complexity of the processes involved. This is supported by the observation that band 3 mutations may also be associated with ovalocytosis or with stomatocytosis (Delaunay, 2007). Since the main trigger for erythrocyte loss in G6PD-deficient erythrocytes is oxidative stress (Beutler, 2008), erythrocytes of affected individuals have been used to investigate the role of oxidation in aging-associated changes in band 3. The results support a connection between hemoglobin denaturation, vesicle formation, oxidation and phosphorylation of band 3 (Minetti et al., 1996; Pantaleo et al., 2011). The latter is in line with the recent awareness of the activity in the erythrocyte of multiple signaling pathways that regulate the interaction of membrane proteins with eachother and with cytosolic proteins. Especially research of the mechanisms that regulate recruitment and activity of kinases and phosphatases is likely to reveal more insight into the processes underlying maintenance and loss of erythrocyte morphology and metabolism in health, disease and aging.

Over the years, data from comparing young with old, and control with pathological erythrocytes, have provided the tools not only to investigate erythrocyte aging in disease, but also to test theories on aging mechanisms *in vitro* (Bosman and Kay, 1988). The latter has led to two main conclusions: 1, investigation of any aging-associated susceptibility to physiological stress-mimicking conditions may reveal the most relevant lesions at the molecular level, and the mechanisms leading to these lesions (Ghashghaeinia et al., 2012); 2, the experimental treatment that results in the highest degree of similarity in morphological, structural, and functional changes to those observed during aging *in vivo*, is storage in blood bank conditions (Bosman and Kay, 1988). Oxidation *in vitro* or reduced protection against oxidative damage *in vitro* and *in vivo* are second best (Bosman and Kay, 1988; Kay et al., 1988a; Burger et al., 2013).

## Blood bank storage, aging, and removal after transfusion

There are many excellent articles and reviews on what happens with the erythrocyte during its sojourn in the blood bank (Hess, 2012). Here we summarize and discuss these events in relation to the aging process *in vivo*, and to their possible effect on their survival after transfusion.

### Lifespan

Erythrocyte survival and lifespan measurements obtained with transfused erythrocytes are in good accordance with those obtained using metabolic labeling studies, indicating an overall good survival with a maximum of 135 days after transfusion (Mollison et al., 1987; Luten et al., 2004). However, almost all studies show the disappearance of 5–10% within the first 24 h after transfusion, which is followed by a linear disappearance curve. This percentage of rapidly disappearing erythrocytes increases with storage time to 25% or more (Luten et al., 2008a). These data suggest that, during their stay in the blood bank, erythrocytes become increasingly vulnerable to as yet unknown stressful conditions they encounter after transfusion. We have postulated that the fraction of quickly disappearing erythrocytes is the main cause of the adverse events of transfusions, especially in transfusion-dependent patients. An overload of the reticulo-endothelial system could lead to hemolysis and the accumulation of neoantigens, triggering iron acccumulation, inflammation and the formation of alloantibodies.

### Volume and density

Blood bank storage affects volume regulation as indicated, among other things, by the increase in erythrocyte volume already in the first week of storage (Luten et al., 2008b). This is most likely due to the far from physiological composition of most storage solutions, and seems to be readily reversible upon incubation in buffers with a pH and ion concentrations similar to those of the blood. As a complicating factor, storage may affect cation transport and volume regulation differently in young and old erythrocytes (Minetti et al., 2001). There is a striking change in erythrocyte shape with storage, apparently progressing from echinocytes and stomatocytes to an irreversible spherocyte morphology (Blasi et al., 2012). Density separation results in cell fractions in which the increase in density is accompanied by an increase in HbA1c, suggesting that this method is, in principle, also suitable for the study of aging *in vitro* (D'Alessandro et al., 2013b). It is tempting to speculate that the more severely and apparently irreversibly deformed erythrocytes comprise at least a fraction of the removal-prone erythrocytes. Cell volume and density are important factors in deformability. Most data indicate that deformability may decrease with storage time, but to an extent that depends heavily on the methods to measure this parameter. The outcomes of most ektacytometry measurements, for example, are likely to be determined by a decrease in the surface/volume ratio, resulting from vesiculation, and a possibly compensatory decrease in the cellular hemoglobin concentration (Cluitmans et al., 2012).

### Vesiculation

The availability of an easily accessible source of relatively pure preparations in the form of erythrocyte concentrates in the blood bank has enabled the generation of many data on storage vesicles (Greenwalt, 2006). However, there is no comprehensive theory on the mechanism of their generation, or on their putative contribution to the adverse side effects of transfusion. Blood bank vesicles are enriched in modified hemoglobin species, as are the erythrocyte-derived vesicles in the plasma (Willekens et al., 2003; Bosman et al., 2008a). Their membrane protein composition, on the other hand, is different from that of erythrocyte-derived vesicles from the plasma, suggesting different vesiculation mechanisms *in vitro* and *in vivo* (Greenwalt, 2006; Bosman et al., 2008b; Kriebardis et al., 2008; Salzer et al., 2008). Also, blood bank vesicles contain more and different plasma-derived proteins, especially immunoglobulins and complement proteins (Bosman et al., 2008b; Kriebardis et al., 2008). The comparison of blood bank vesicles with plasma vesicles is complicated by their accumulation over time in the blood bag, as this results in a heterogeneity in vesicle age that is likely to be much higher than those of freshly isolated vesicles. Also, there is a distinct possibility that the mechanism of vesiculation changes with storage time and storage medium (Greenwalt and Dumaswala, 1988; Bosman et al., 2010a; Sparrow et al., 2013). A more extensive study especially of the biological activity of blood bank vesicles is warranted by their biological activity, and likely effect on the circulation and immune system of the patient (Gould et al., 2007; Donadee et al., 2011; Kozuma et al., 2011; Xiong et al., 2011).

### Removal signals and mechanisms

Band 3 is very sensitive to proteolytic breakdown, as becomes apparent not only upon immunoblot analysis of purified erythrocytes kept in physiological buffer solutions, but also when stored as whole blood or during storage in blood bank conditions. This sensitivity is most apparent using antibodies against parts of the cytoplasmic domain, suggesting that this domain is especially vulnerable (Bosman et al., 2008a). The changes in this domain are likely to affect the connection between lipid bilayer and the cytoskeleton, and the binding of key enzymes of the glycolysis. Thus, early changes in the cytoplasmic domain may very well be responsible for the storage-associated changes in erythrocyte morphology and metabolism. On the other hand, new metabolomic data support the hypothesis that storage exacerbates the effect of naturally occurring oxidative stress by disturbing the physiological balance between glycolysis, pentose phosphate pathway, and glutathione homeostasis (Gevi et al., 2012; Rinalducci et al., 2012). The apparent reversibility of these changes, especially within the first weeks of storage, may be explained by the redundancy of available binding sites. There are much more band 3 than ankyrin molecules, and the dynamic equilibrium between band 3 monomers, dimers and tetramers may compensate for a small decrease in ankyrin-binding (or enzyme-binding) sites.

The number of IgG molecules that is bound per erythrocyte only slightly increases with storage time. Even after the maximal storage period, the percentage of erythrocytes with an amount of IgG that is sufficient for recognition by macrophages is still very small (Kay, 2005). It is noteworthy that the binding of IgG increases within the first weeks of storage, and then decreases again (Luten et al., 2004; Dinkla et al., 2012a). We speculate that this may be caused by the lysis of the very old erythrocytes early in the storage period, leading to association of spectrin or actin to other, intact cells. This could trigger the binding of low-affinity antibodies, that are normally present in the blood (Garratty, 2005). The number of IgG-containing erythrocytes at the end of the maximal storage time, even after incubation with autologous plasma, is much lower than predicted if the aging process *in vitro* would proceed in an identical manner and with the same speed as it does *in vivo*. If the latter scenario, the percentage of senescent cell antigen-exposing (and thus IgG-containing) erythrocytes would be much higher than observed, even when corrected for the difference in temperature. Therefore, we must conclude that storage in blood bank conditions may have some molecular features of aging *in vivo* (see also Figure 1), but that the processes underlying the fast removal of up to 30 percent of the erythrocytes after transfusion cannot be ascribed to a physiological aging process. The recently described, storage-associated increase in the binding of IgG from patients with autoimmune hemolytic anemia to blood bank erythrocytes, shows that aging *in vitro* may, in certain conditions, assume a pathological form (Dinkla et al., 2012a).

Similarly, the very small increase in the number of PS-exposing erythrocytes during storage is far from sufficient to explain the fast removal of up to 30 percent of the stored erythrocytes after transfusion (Verhoeven et al., 2006; Bosman et al., 2011). However, with storage the erythrocytes become very sensitive to stress-induced PS exposure, especially to the near-physiological stress consisting of incubation in a hyperosmotic buffer (Bosman et al., 2011). Together with a concomitant storage-associated increase in susceptibility to generate vesicles (Burger et al., 2013), this observation emphasizes the predisposing, “sublethal” nature of the events that occur in the blood bank.

In a probably analogous manner, there is a detectable change in CD47 conformation during storage only after transfusion *in vitro*, i.e., after incubation of the stored erythrocytes with whole blood (Burger et al., 2012). Also, storage is associated with an increased sensitivity to a band 3 ligand-induced binding of autologous IgG (Bosman et al., 2010b). The storage-associated increase in sensitivity to lipase-induced morphological and biochemical alterations points toward a hitherto little-studied involvement of lipids and lipid organization in erythrocyte vesicle formation and antigen presentation (Salzer et al., 2008; Dinkla et al., 2012b). In general, parameters for a “sublethal injury” such as these are likely to carry more physiologically and clinically relevant weight than osmotic or mechanical fragility (Raval et al., 2010, 2013; Cluitmans et al., 2012).

## Conclusions

The presently available data show that neither our knowledge of the identity of the molecules that signal removal of physiologically aged erythrocytes, nor of the identity of the mechanisms by which they are generated, has progressed much in the last two decennia. One notable exception is the incorporation of vesicle formation in the aging process *in vivo* (Willekens et al., 2008). This is in spite of the strong increase in the number of the phenomena described to accompany especially erythrocyte aging *in vitro*. The recent increase in the number of data on the presence and activity of signaling pathways regulating erythrocyte function, morphology and metabolism, mostly resulting from disease-centered research, opens new possibilities to unravel the mechanisms involved in erythrocyte aging *in vivo* as well as *in vitro*. Regarding the latter, all present data show that storage may be the best available model for studying erythrocyte aging, but do not support the theory that storage in the blood bank is an accelerated form of physiological aging or of a pathological form of aging *in vivo*. This supports the statement that there is still a poor understanding of the storage lesion and its effect on erythrocyte performance. In the view presented here, the most recent data indicate that the processes that occur during storage render the erythrocytes especially vulnerable to the aging phenotype-inducing conditions they encounter after transfusion in the circulation. The increase in susceptibility to stress-induced PS exposure and shrinkage that also occurs during aging *in vivo* (Ghashghaeinia et al., 2012), provides a functional connection between aging *in vitro* and aging *in vivo*. In this context, a recent definition of aging as a “de-tuning of adaptation with increasing age” seems to be directly applicable at the cellular level (Raval et al., 2013).

This leads to the practical conclusion that future research on improving erythrocyte survival after transfusion should concentrate on relating the changes, observed in erythrocytes during storage, to their resistance to physiological stress that induces the appearance of removal signals after transfusion. This stress could be mechanical, e.g., as experienced during passage through the microcapillaries and the spleen, but also biochemical, e.g., as occurring during the gradual exhaustion of the defense against oxidation and oxidation-induced protein denaturation. Together with the available knowledge on the identity of removal signals, and the identification of the relevant signaling pathways that is likely to happen in the near future, this approach provides a sorely needed, experimental platform to investigate the mechanisms responsible for the dangerous, fast removal of a considerable fraction of the blood bank erythrocytes early after transfusion.

### Conflict of interest statement

The author declares that the research was conducted in the absence of any commercial or financial relationships that could be construed as a potential conflict of interest.
